# Seismic and Restoration Assessment of Monumental Masonry Structures

**DOI:** 10.3390/ma10080895

**Published:** 2017-08-02

**Authors:** Panagiotis G. Asteris, Maria G. Douvika, Maria Apostolopoulou, Antonia Moropoulou

**Affiliations:** 1 Computational Mechanics Laboratory, School of Pedagogical and Technological Education, Heraklion, 14121 Athens, Greece; mariadouvika7@gmail.com; 2 Laboratory of Materials Science and Engineering, School of Chemical Engineering, National Technical University of Athens, 15780 Athens, Greece; mairi_apostol@hotmail.com (M.A.); amoropul@central.ntua.gr (A.M.)

**Keywords:** historical structures, fiber-reinforced mortars, fragility curves, masonry structure, restoration, structural assessment, structural modelling

## Abstract

Masonry structures are complex systems that require detailed knowledge and information regarding their response under seismic excitations. Appropriate modelling of a masonry structure is a prerequisite for a reliable earthquake-resistant design and/or assessment. However, modelling a real structure with a robust quantitative (mathematical) representation is a very difficult, complex and computationally-demanding task. The paper herein presents a new stochastic computational framework for earthquake-resistant design of masonry structural systems. The proposed framework is based on the probabilistic behavior of crucial parameters, such as material strength and seismic characteristics, and utilizes fragility analysis based on different failure criteria for the masonry material. The application of the proposed methodology is illustrated in the case of a historical and monumental masonry structure, namely the assessment of the seismic vulnerability of the Kaisariani Monastery, a byzantine church that was built in Athens, Greece, at the end of the 11th to the beginning of the 12th century. Useful conclusions are drawn regarding the effectiveness of the intervention techniques used for the reduction of the vulnerability of the case-study structure, by means of comparison of the results obtained.

## 1. Introduction

There are many historic monuments of high architectural and cultural value around the world that deserve protection against major earthquakes. The purpose of investigating the seismic behavior of ancient monuments such as masonry structures is two-fold: (1) to identify the mechanisms that have allowed the surviving monuments to avoid structural collapse and destruction during strong earthquakes (in the course of history) and (2) to select suitable and effective rehabilitation techniques.

The majority of historical and monumental structures consist mainly of masonry material (which is considered to be the historically oldest structural material), and they are mainly located around the Mediterranean Sea, which is a geographical region subjected to a higher risk of earthquakes than other parts of the world. These two characteristics, namely the masonry material and the seismic location, delineate the framework for any restoration scenarios. It is worth mentioning that masonry material is a composite, multiphase material that exhibits a distinct brittle and anisotropic nature. The strongly anisotropic nature of masonry is based on the fact that the mortar joints act as planes of weakness, as well as on the nature and structure of mortar and brick material. Especially in ancient times, the construction of monumental structures employed the salient technique of using fibers as reinforcement. The timeline of using fibrous materials in construction is nearly as long as the history of construction itself. As is historically documented, horsehair was used in mortar and straw in mudbricks. This ancient reinforcement technique significantly contributes to the anisotropic nature of masonry material.

The above-mentioned aspects are of great interest for engineering practice, as well as for educational curriculum in engineering faculties. Furthermore, the need for multidisciplinary cooperation based on the principles imposed by past or current regulations and scientific charters (e.g., the Athens Charter 1931 (International council on monuments and sites, ICOMOS 1931), the Venice Charter 1964 (ICOMOS 1964), etc.) [1,2] makes the entire process of analysis even more demanding.

Our research has adopted the core values embedded in the international standards, as delineated by the principles of research and documentation, authenticity and integrity, compatibility (being, at the same time visual and physical and/or chemical), minimal intervention and the degree of reversibility (as it is rare to achieve a fully-reversible technique). The knowledge of experts’ work dealing with the modeling, the assessment of seismic vulnerability and the restoration techniques provides an essential input to this effort. Detailed and in-depth state-of-the-art reports can be found in [3,4,5,6,7,8,9,10,11,12,13,14,15,16,17,18,19,20,21,22,23]. In particular, regarding the development of rehabilitation mortars, the state-of-the-art research works on Roman and Byzantine mortars in [24] should be taken into account.

The present work describes the detailed methodology for estimating the seismic vulnerability of masonry monumental structures, as applied to the estimation of the vulnerability and optimal renovation scenario for the Kaisariani Monastery’s byzantine church, which was built in Athens, Greece, at the end of the 11th to the beginning of the 12th century. Emphasis has been placed on determining the construction failures by means of robust simulations and in designing the composition of rehabilitation mortars, which are ranked according to their reduction of seismic vulnerability, thus leading to the selection of the optimal intervention material.

## 2. Proposed Methodology

**Figure 1:**
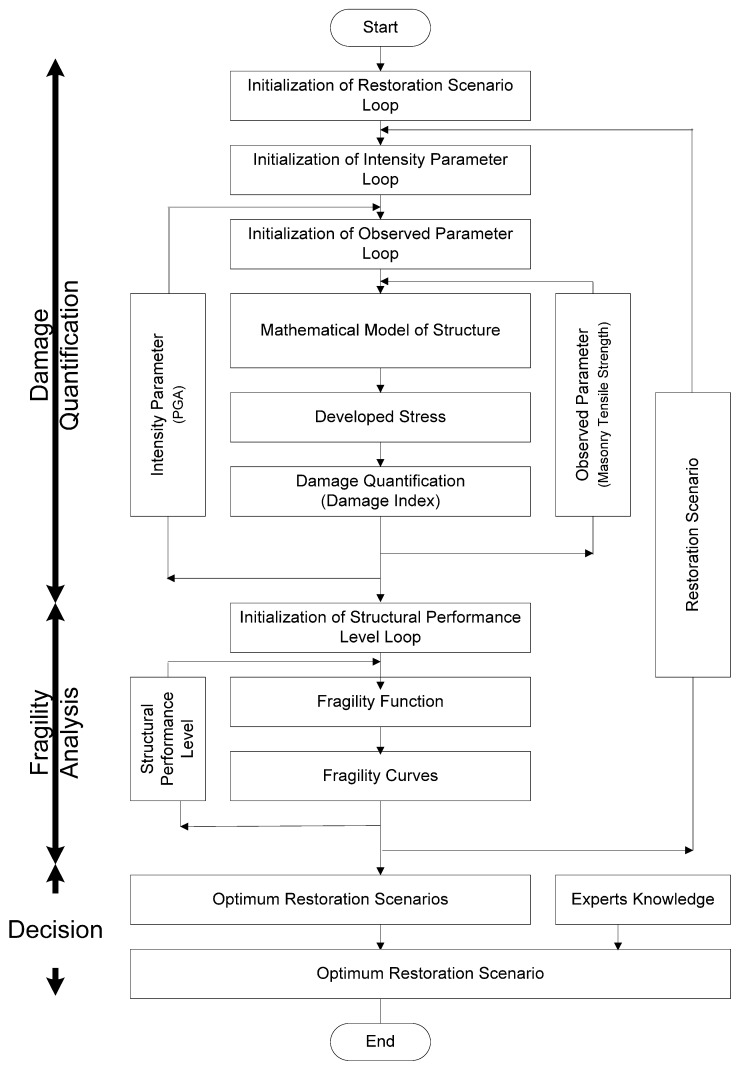
Flowchart depicting the applied methodology for vulnerability and restoration assessment. PGA, peak ground acceleration.

In the framework of the above-mentioned scientific charters and classical state-of-the-art reports the herein proposed methodology (Figure 1) consists of the following ten distinct steps:

Step 1: Historical and experimental documentation

There are certain aspects that should be followed before carrying out a rigorous structural analysis. In particular, experience shows that the structural analysis regarding the seismic response of a monument is an integral part of the broader study of the monument; the history and architecture of the monument are indispensable prerequisites for the structural analysis, in order to account for all initial and consecutive construction phases, previous interventions or additions, etc. Furthermore, the results of the experimental investigations regarding geometrical data, the in situ evaluation of the strength of materials, the structural properties of masonry walls, the dynamic response of the construction, as well as the results of possible previous monitoring can be crucial for reliable modeling and a successful assessment of a masonry monumental structure.

Detailed and in-depth state-of-the-art reports on the historical and experimental documentation can be found in [5,7,9,11,12,14,25,26,27].

Step 2: Material characteristics

The characteristics of materials composing the structure comprise basic input data for a reliable and robust structural modeling of the structure. Namely, the compressive/tensile strength of the materials, the modulus of elasticity and Poisson ratio are of primary importance, at least as far as a linear/elastic analysis is concerned. For the estimation of those parameters, the combination of analytical or semi-empirical methods and experimental data (both in situ and in vitro) has to be used. For the determination of the masonry compressive and tensile strength, several semi-empirical expressions are available in the literature. In the majority of these expressions, global effects contributing to the system resistance, such as buckling effects or local compression resistance, are not considered. Detailed and in-depth state-of-the-art reports on the estimation of masonry strength can be found in [28,29,30,31,32].

For the estimation of the compressive and tensile strength of masonry, a plethora of formulae has been proposed. For the special case of low-strength stone-masonry, with a single leaf, the strengths can be estimated [33] by Equations (1) and (2):

(1) $$f_{wc}=\xi \left[\left(\frac{2}{3}\sqrt{f_{bc}}-\alpha \right)+\beta \;f_{mc}\right]\;(\text{in MPa})$$

(2) $$f_{wt}=\frac{2}{3}f_{mt}$$

where f_*wc*_, f_*wt*_ are the compressive and tensile strength of the masonry respectively, f_*mc*_, f_*mt*_ are the compressive and tensile strength of the mortar, respectively, and f_*bc*_ is the compressive strength of the block/stone material. *α* is a reduction factor due to the non-orthogonality of blocks (*α* = 0.5 for block stones and *α* = 2.5 for rubble stones). *β* is a mortar-to-stone factor (*β* = 0.5 for rough stones and *β* = 0.1 for very smooth-surface stones). *ξ* is a factor expressing the adverse effect of thick mortar joints, *ξ* = 1/[1 + 3.5(k − k_o_)] (k = volume of mortar/volume of masonry) and k_o_ = 0.3.

Detailed and in-depth state-of-the-art reports on the mechanical characteristics of masonry material, including two- and three-leaf stone masonry, can be found in [9,33,34].

Step 3: Structural model

The simplest approach to the modeling of complex historic buildings is given by the application of different structural elements, employing truss, beam, panel, plate or shell elements to represent columns, piers, arches and vaults, with the assumption of homogeneous material behavior.

A 3D finite element model (with elastic materials), as used in this study, seems to be the most suitable for the analysis, at least as far as a global assessment is concerned. For higher model reliability, specific simulation parameters, such as the rotation capacity of the wooden floor or roof connection with the masonry wall, the degree of connections between intersected walls, the influence of spandrel beams, etc., must be taken into account.

Step 4: Actions

Different loading cases have to be taken into consideration, including seismic actions for structures built in seismic areas. Combinations of dead loads, live loads and earthquake demands, have to be used. The earthquake has to be considered along all unfavorable directions for the building. Nevertheless, certain issues are still open, regarding, e.g., the poor hysteretic behavior of masonry or the adverse influence of the simultaneous vertical component of the seismic action.

Step 5: Analysis

Using input data of the previous steps, a finite element analysis is performed and stresses (normal-shear), displacements at the joints of the mesh, are calculated. Due to the actual behavior of plain masonry and the high degree of uncertainty in the previous steps, elastic analysis is a primary valuable tool to be used for such structures, especially before any repair and/or strengthening.

Step 6: Failure criterion and damage indices

A failure criterion must be established for the definition of the damaged regions of the structure (as a first insight). Taking into account the conclusions of Step 2 concerning materials’ characteristics, such a criterion is proposed and will be used as an input to carry out the analysis.

These failure results are used as input data for the development of a damage index. Based on this index, the possibility of a structure being damaged beyond a specified level (which can be defined as heavy, moderate, insignificant) for various levels of ground acceleration is determined. This information is important during the analysis and redesign process for a historical structure since it gives the opportunity to investigate different scenarios with different options regarding repair/strengthening.

Step 7: Seismic vulnerability assessment

Based on the damage indices computed in the previous step, a quantification of the seismic vulnerability should be achieved at this step. For the assessment of the capacity of the structure, many techniques have been proposed. Taking into account that a plethora of the parameters involved in the modeling of a structure exhibits a probabilistic nature (e.g., materials characteristics and loadings (seismic excitations)), the most suitable, reliable and robust technique for masonry structure is considered to be a probabilistic assessment, which can be achieved through fragility analysis.

Step 8: Repairing and/or strengthening decisions and reanalysis

According to the results of Steps 5 and 6, all of the damaged regions are repaired and/or strengthened. The method to be used, the extent of the interventions, the type of the materials, etc. could be directly related to the results and are based on semi-empirical expressions for the final mechanical characteristics of the masonry.

It should be noted that due to the restrictions imposed by scientific charters, the readily-available, efficient and eligible rehabilitation instrument is the use of traditional rehabilitation mortars, the composition of which is determined, to a high degree, by the composition of the mortars of the existing structure. If previous interventions have taken place, the composition of the mortars must be revealed by means of the detailed characterization of the mortars of the initial construction if they are physically available or by searching historical sources if they are not physically available. Although this is, most of the times, a particularly cumbersome process, it is nevertheless an absolutely essential process for the successful repairs and renovation of the monument (as well as for the training and education process).

Finally, a new structural analysis has to be performed including all of the final materials, loading and structural data. Results of the analysis have to be used subsequently in the processes of Steps 5 to 7, leading to a final approval (or rejection) of the decisions already made for the repair or strengthening of the existing structure.

Step 9: Final decision about the most suitable and effective restoration scenario

At this point, it should be mentioned that the final decision for the optimum restoration scenario must be made by a consensual decision-making procedure encompassing the full spectrum of the experts who represent the knowledge disciplines contributing to the solution of the problem at hand. They should take into account the ranking of the effective restoration scenario, based on the proposed methodology, feeling free to investigate a number of ranked scenaria without being obliged to select just the one with the top ranking. The deterministic ranking of the restoration scenario is a useful technique; however, the collective expertise of the experts, which might not be embedded in full in the deterministic scenaria, is considered to be a very significant factor (even more significant than the deterministic rankings) toward a successful proposal for a restoration, even more so if a monument restoration is at hand.

Step 10: Explanatory report

The last step, as a result of the proposed methodology, includes the detailed ‘explanatory report’, where all of the collected information, the diagnosis, including the safety evaluation, and any intervention decisions should be fully detailed. This document is essential for eventual future analyses and intervention measures in the structure.

## 3. Failure Criteria

A key point for a successful application of the proposed methodology is the use of a reliable failure criterion for the modelling of the masonry failure. To this end, a tensor polynomial has been used for the modeling of the masonry failure. Specifically, according to this criterion, the masonry material is assumed to exhibit a distinct anisotropic nature, and the failure surface can be described by the following equation:

(3) $$f\left(\sigma \right)=F_{i}\sigma_{i}+F_{\mathrm{ij}}\sigma_{i}\sigma_{j}+F_{\mathrm{ijk}}\sigma_{i}\sigma_{j}\sigma_{k}+\cdot \cdot \cdot \{\begin{array}{ll} <1 & \mathrm{no}\;\mathrm{failure}\\ =1 & \mathrm{failure}\\ >1 & \mathrm{exceeded}\;\mathrm{failure} \end{array}$$

where i, j, k = 1, 2, ..., 6. F_i_, F_ij_ and F_ijk_ are (strength) tensors of the second, fourth and sixth rank, respectively.

Based on the above equation, restricting the analysis to a plane stress state, assuming that a cubic formulation is a reasonably accurate representation of the failure surface and by taking into consideration the symmetry and anisotropic nature of the material [35,36,37], the masonry failure surface, known as the cubic tensor failure criterion, can be expressed by the following equation:

(4) $$\begin{array}{r} f\left(\sigma_{x},\sigma_{y},\tau \right)=2.27\sigma_{x}+9.87\sigma_{y}+0.573\sigma_{x}^{2}+1.32\sigma_{y}^{2}+\;6.25\tau^{2}-0.30\sigma_{x}\sigma_{y}+\\ 0.009585\sigma_{x}^{2}\sigma_{y}+\;0.003135\sigma_{x}\sigma_{y}^{2}+0.28398\sigma_{x}\tau^{2}+\;0.4689\sigma_{y}\tau^{2}=1 \end{array}\}$$

or by using the dimensionless terms $\left({\bar{\sigma }}_{x}=\frac{\sigma_{x}}{f_{wc}^{90{}^{\circ}}},\;{\bar{\sigma }}_{y}=\frac{\sigma_{y}}{f_{wc}^{90{}^{\circ}}},\;\bar{\tau }=\frac{\tau }{f_{wc}^{90{}^{\circ}}}\right)$ and taking into account that $f_{wc}^{90{}^{\circ}}=7.555\;Mpa$, the above equation can be written in the form of:

(5) $$\begin{array}{l} 17.15\;{\bar{\sigma }}_{x}+74.57\;{\bar{\sigma }}_{y}+32.71\;{\bar{\sigma }}_{x}^{2}+75.34\;{\bar{\sigma }}_{y}^{2}+\;356.74\;{\bar{\tau }}^{2}+\\ -17.12\;{\bar{\sigma }}_{x}{\bar{\sigma }}_{y}+4.13\;{\bar{\sigma }}_{x}^{2}\;{\bar{\sigma }}_{y}+1.35\;{\bar{\sigma }}_{x}\;{\bar{\sigma }}_{y}^{2}+122.46\;{\bar{\sigma }}_{x}{\bar{\tau }}^{2}+\\ +202.20\;{\bar{\sigma }}_{y}{\bar{\tau }}^{2}=1 \end{array}\}$$

Eliminating all third-order terms in (5), a simplified failure criterion is derived [35,37]:

(6) $$\begin{array}{l} 17.15\;{\bar{\sigma }}_{x}+74.57\;{\bar{\sigma }}_{y}+32.71\;{\bar{\sigma }}_{x}^{2}+75.34\;{\bar{\sigma }}_{y}^{2}+\;356.74\;{\bar{\tau }}^{2}\\ -25.91\;{\bar{\sigma }}_{x}{\bar{\sigma }}_{y}=1 \end{array}\}$$

**Figure 2:**
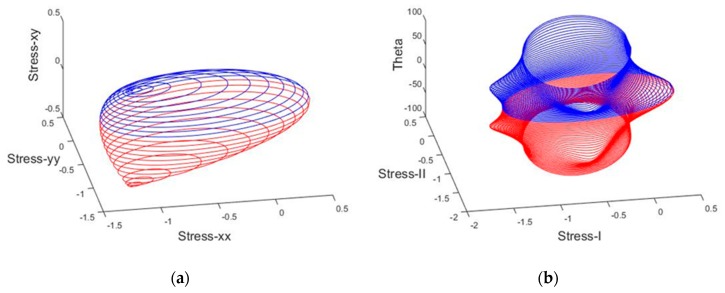
Non-dimensionless failure surface of masonry material using the cubic tensor polynomial failure criterion: (**a**) non-dimensionless normal stress terms; (**b**) non-dimensionless principal stress terms.

**Figure 3:**
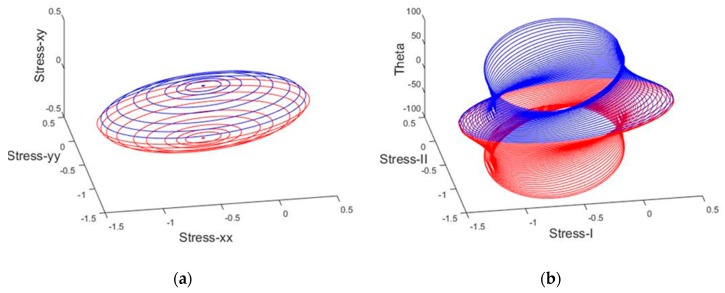
Non-dimensionless failure surface of masonry material using the simplified failure criterion: (**a**) non-dimensionless normal stress terms; (**b**) non-dimensionless principal stress terms.

This simple form of the criterion has already been used by [35,38,39,40,41,42]. In Figure 2 and Figure 3, the graphical representations of the cubic and simplified failure criterion in non-dimensionless principal and normal stress terms are depicted.

At this point, it is of great interest to know the minima and the maxima of the functions that express the above failure criteria (surfaces). This can be achieved by equating to zero the three partial derivatives of the failure criterion functions with respect to the three variables, ${\bar{\sigma }}_{x}$, ${\bar{\sigma }}_{y}$ and $\bar{\sigma }$. Namely,

(7) $$\frac{\partial f}{\partial {\bar{\sigma }}_{x}}=0,\frac{\partial f}{\partial {\bar{\sigma }}_{y}}=0\;\mathrm{and}\;\frac{\partial f}{\partial \bar{\tau }}=0$$

The minimum and maximum values for each failure criterion, across each one of the axes, are presented in Table 1. It is worth mentioning that the maximum dimensionless value of the shear strength based on the cubic failure criterion is almost double compared to the corresponding value in the simplified failure criterion (0.50 instead of 0.27, respectively). Based on this finding, the type and the percentage of the failure will differ significantly for a structure that depicts shear sway behavior compared to a structure with a cantilever bending behavior.

## 4. Damage Index

Quantifying damage control in a building is a complex task, especially under seismic action. There are several response parameters that can be instrumental in determining the level of damage that a particular structure suffers during ground motion; the most important ones are: deformation, relative velocity, absolute acceleration and plastic energy dissipation (viscous or hysteretic).

Controlling the level of damage in a structure consists primarily of controlling its maximum response. Damage indices establish analytical relationships between the maximum and/or cumulative response of structural components and the level of damage they exhibit [43]. A performance-based numerical methodology is possible if, through the use of damage indices, limits can be established for the maximum and cumulative response of the structure, as a function of the desired performance of the building for the different levels of the design ground motion. Once the response limits have been established, it is then possible to estimate the mechanical characteristics that need to be supplied to the building, so that its response is likely to remain within the limits.

For the case of masonry structures, a new damage index has been proposed by Asteris [13], employing as the response parameter the percentage of the damaged area of the structure relative to the total area of the structure. The proposed damage index (DI) for a masonry structure can be estimated by the following Equation (8):

(8) $$[DI]=\frac{A_{fail}}{A_{tot}}\times 100$$

where *A_fail_* is the damaged surface area of the structure and *A_tot_* the total surface area of the structure.

## 5. Structural Performance Levels

As practiced today, performance-based seismic design is initiated with an interplay between demands and appropriate performance objectives. The engineer then has to develop a design capable of meeting these objectives. Performance objectives are expressed as an acceptable level of damage, typically categorized as one of several performance levels, such as immediate occupancy, life safety or collapse prevention, given that ground shaking of a specified severity is experienced.

In the past, the practice of meeting performance-based objectives was already included in design practice, but in a rather informal, simplistic and non-standard way. Some engineers would characterize performance as life-safety or not; others would assign ratings ranging from poor to good. This qualitative approach adopted for performance prediction was appropriate given the limited capability of seismic-resistant design technology to deliver building designs capable of quantifiable performance.

We consider three structural performance levels: (a) heavy damage; (b) moderate damage and (c) insignificant damage, in a similar way to the Federal Emergency Management Agency (FEMA 273 1997) [44]. The performance levels are defined by the values of DI (as shown in Table 2). A value of (DI) less than 15% can be interpreted as insignificant damage; from 15% to less than 25%, as moderate damage; and greater than or equal to 25% as heavy damage. In fact, other approaches could be used, according to the recent European Codes (EC8 2005) [45], based on a more engineered (and more detailed) estimation of damage.

## 6. Fragility Analysis

One of the problems the engineer must face and resolve at later stages of the global assessment involves the quantitative vulnerability assessment of the building as it is (damaged or not), as well as its behavior once “modified” after interventions. In other words, a method is necessary to assess the seismic vulnerability of the existing structure, as well as to assess the intervention scenarios and rank them according to the reduction of the seismic vulnerability, thus leading to the selection of the optimal one. One of the most important tools is considered to be fragility analysis, which provides a measure of the safety margin of the structural system above specified structural performance/hazard levels.

A number of methodologies for performing fragility analysis has been proposed in the past (used to assess the behavior of structural systems). Simplified methodologies for fragility evaluation have been proposed in [46].

Evaluating seismic fragility information curves for structural systems involves: (a) information on structural capacity and (b) information on the seismic hazard. Due to the fact that both the aforementioned contributing factors are uncertain to a large extent, the fragility evaluation cannot be carried out in a deterministic manner. A probabilistic approach, instead, needs to be utilized in the cases in which the structural response is evaluated and compared against “limit states”, that is the limiting values of the response quantities correlated to structural damage.

**Figure 4:**
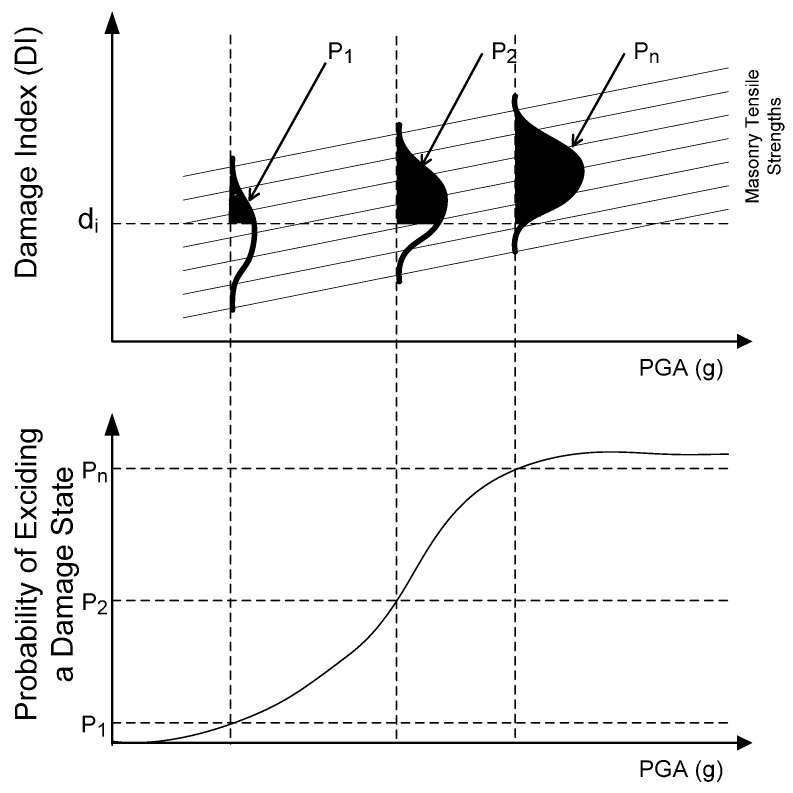
Development process of analytical fragility curves.

Fragility, as it is shown in Figure 4, is the probability of the structural damage reaching or exceeding a certain damage threshold di (performance level) under a given earthquake level (peak ground acceleration (PGA)). It generally increases as the earthquake intensity level increases. The failure domain is where the damage index (DI) overcomes a specified threshold (damage index or performance level).

Fragility is evaluated as the total probability of a response parameter R exceeding the allowable response value *r*_lim_ (limit-state), for various earthquake intensities *I*. In mathematical form, this is simply a conditional probability [47,48] given by the following Equation (9):

(9) $$Fragility=P\left[R\geq r_{\lim}|I\right]=\sum_{j}^{3}P\left[R\geq r_{\lim}|I,C\right]\;P\left(C=c_{j}\right)$$

where *P*(*C* = *c_j_*) is the probability that capacity *c_j_* occurs. In the following examples, the basic steps for the development of the fragility curves are briefly presented.

## 7. Case Study

In this section, the reliability, the effectiveness and the robustness of the methodology, for the assessment of the seismic vulnerability of a monumental masonry structure, are presented through a step-by-step approach. In particular, the proposed methodology has been applied to a historical and monumental masonry structure in Athens, Greece.

### 7.1. Identification of the Structure

**Figure 5:**
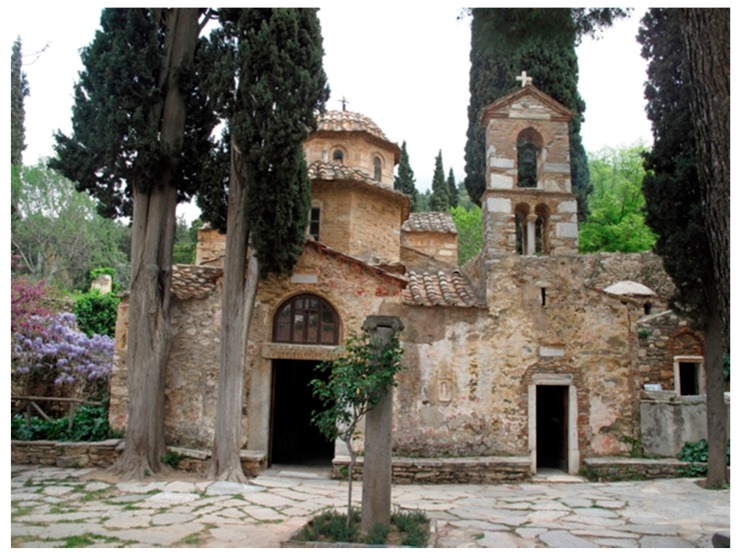
Front façade of the Kaisariani monastery.

**Figure 6:**
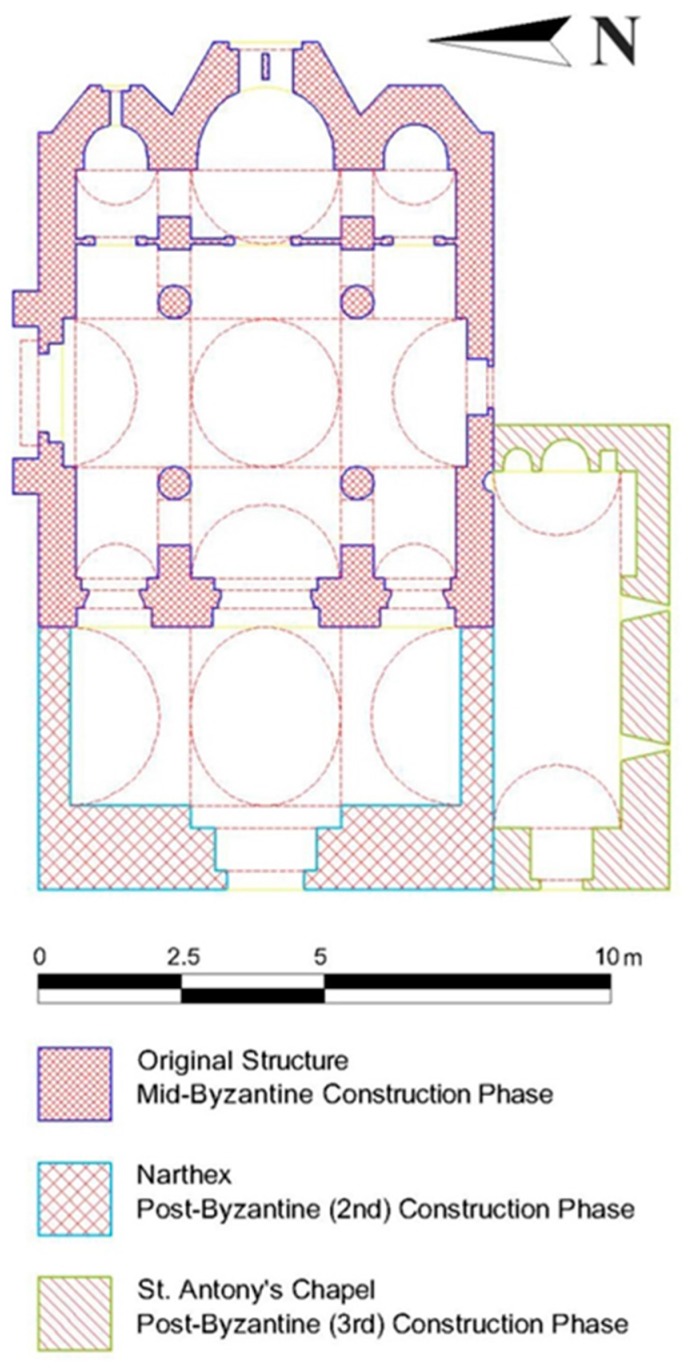
Plan view of the Kaisariani monastery.

The structure under investigation is the Kaisariani Monastery, a byzantine church (Figure 5 and Figure 6) that was built in Athens, Greece, at the end of the 11th to the beginning of the 12th century. Moreover, the site has a far longer history as a cult center: in Antiquity, it was probably a site dedicated to Aphrodite before being taken over by Christians in the 5th/6th centuries. The remains of a large early Christian basilica lie to the west, over which a smaller church was built in the 10th/11th centuries.

The whole structure is composed of three different and distinct individual constructions, which have been constructed in three different time periods. Namely, the original mid-byzantine structure, which was built at the end of the 11th to the beginning of 12th century; this structure is a complete symmetry cross-in-square four-column domed church, without a narthex; the second construction phase was in the early 17th century where the domed narthex was added; and the third construction phase is related to the construction of the St. Antoine chapel in the late 17th century (Figure 6).

### 7.2. Characterization and Classification of Materials

Various lab techniques were applied in order to characterize and study the samples taken from the Catholicon of the Kaisariani Monastery [22]. Fiber optic microscopy (FOM) was employed in order to examine the samples microscopically, using a (PICO SCOPEMAN-MORITEX). Sieve analysis was performed according to Normal 27/88 [49] using sieves according to ISO 565 [50]. Differential thermal and thermo-gravimetric analysis (DTA-TG) provided qualitative and quantitative information regarding the composition of the samples (Mettler Toledo 651e). The temperature range applied was 25–1000 °C, and the heating rate was selected at 10 °C/min [24]. X-ray diffraction (XRD) provided information regarding the mineralogical composition of the materials (Advance D8 Diffractometer, Bruker Corporation, Dresden, Germany) [24,49,51]. The microstructural characteristics of the samples were studied through the use of mercury intrusion porosimetry (MIP) with the use of a Pascal 400 porosimeter, Thermo-Electronics Corporation, Milan, Italy [24,49,52]. Furthermore, Schmidt hammer tests were conducted in situ in order to estimate the compressive strength of the brick and stone elements of the cloisonné, using an N-type Schmidt hammer, manufactured by Proceq [53,54].

#### 7.2.1. Characterization and Classification of Historical Mortars

**Figure 7:**
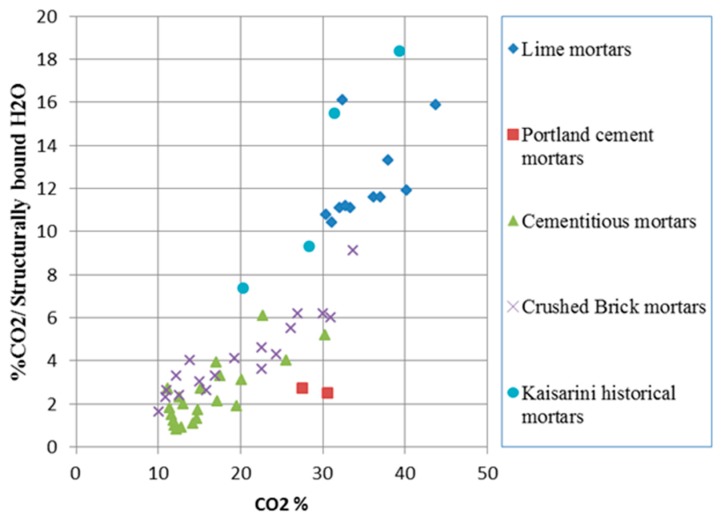
Grouping of the Kaisariani mortars according to thermal analysis results.

**Figure 8:**
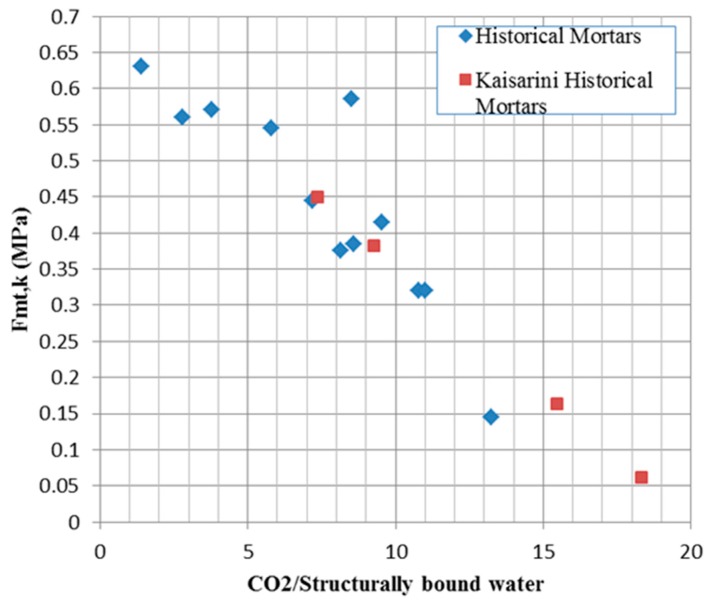
Correlation of tensile strength and inverse hydraulicity index.

The mortars of the Kaisariani Monastery Catholicon are typical lime mortars mixed with calcite and aluminosilicate aggregates, with high porosity values and occasionally the addition of straw or fiber admixtures. In Figure 7, the classification of the mortars is achieved via correlation of the thermal analysis results conducted, in accordance with [55]. The same researchers noted the correlation of historical mortars’ tensile strength with their inverse hydraulicity index, as estimated from thermal analysis results. The inverse hydraulicity index is the ratio of the percentage of CO_2_ mass loss attributed to the decomposition of CaCO_3_ to the percentage of mass loss of structurally-bound water attributed to the dehydration of hydraulic compounds and inversely expresses the level of mortar hydraulicity [56]. Thus, the tensile strength of the historical mortars can be estimated (Figure 8, Table 3).

#### 7.2.2. Mechanical Strength of Building Stones and Bricks

The rebound number (R) was utilized in order to estimate the compressive strength of different building materials, through the use of the instrument’s conversion curves and in accordance with ASTM C 805 [57]. The average rebound number (R) and its standard deviation, as well as the compressive strength that is estimated through the use of the instrument’s conversion curves for the brick and stone elements of the east façade of the cloisonné are stated in Table 4 and Table 5, respectively. The stones were hand-carved and the bricks were handmade, therefore lacking standardization in size. The carved stones ranged in size; however, most were 20 cm in height and 50 in length. The bricks’ dimensions ranged from 2.5 to 5 cm in height and about 20 to 50 cm in length. The shape of the carved stones and the bricks was relatively orthogonal.

There are certainly limitations to the correlation of the average rebound number (R) and compressive strength when applied to historical materials. These limitations are attributed to the lack of correlation standardization and to the sometimes low compressive values of historical materials, in relation to concrete for which the correlations have been realized. Furthermore, surface crusts as degradation products, salts deposition, lack of homogeneity and deteriorated surfaces are only a few of the parameters affecting the uncertainty of the correlation. However, in the case of historical structures, the estimation of the mechanical strength of building elements is important, as sampling is often limited, and adequate samples of proper size and geometry cannot be acquired.

### 7.3. Structural Modeling

**Figure 9:**
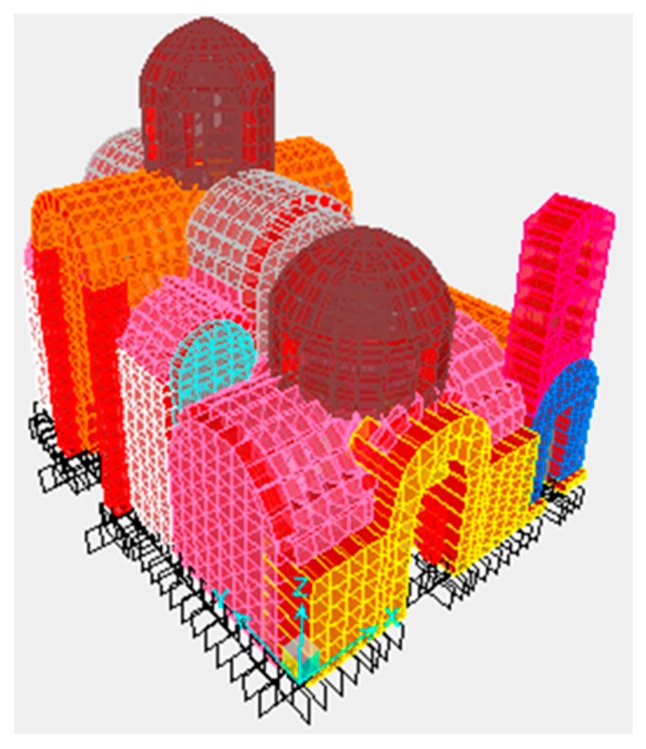
Finite element modeling of the Kaisariani monastery.

The program used to simulate the structure is the software SAP2000 v14 Nonlinear (Walnut Creek, CA, USA). With this software, an appropriate FEM model to calculate the response of the structure was formed. The development of the finite elements mesh was such that the ideal concentration of masses at the nodes simulates well the real mass distribution. This ensures a reliable simulation of the inertial loads for dynamic analysis. To fully determine the deformation of the system, six degrees of freedom for each node were considered. The six degrees of freedom correspond to three translations, along the axes *x*, *y* and *z*, and three rotations of vectors, parallel to the same axes. The model of the building is shown schematically in Figure 9.

The geometrical simulation was done by isotropic surface members (shell elements) and isotropic linear members (frame elements), which are considered to represent with sufficient reliability the properties of the real body. The model used to analyze the building is spatial. The discretization of the finite element mesh was through flat quadrilateral and triangular elements. Depending on the geometry and loading conditions prevailing at each region of the model, the condensation of the data was chosen. In this way, the anisotropic behavior of the masonry structure was better simulated. Specifically, FE mesh condensation occurred on the following areas: locations of concentrated loads, perimeter of the openings, corner areas (wall compounds). For the simulation model, 3.548 nodes, 3.445 surface elements with an average dimension of 0.40 m and 120 beam elements were used.

### 7.4. Seismicity of the Location Area

It is well known that Greece is one of the most seismically-active countries in the world and the most active in Europe. The long documented seismic history of Greece reports many catastrophes due to earthquakes. It could be stated that it is an “ideal seismological laboratory” for the structural engineer. Namely, earthquakes in Greece are strongly related to everyday life, throughout the country’s course in history. The strong earthquakes that have occurred in this, relatively limited, area of the eastern Mediterranean have affected the history, tradition, religion, arts, building habits, political, social and economic status for a very long time. Five percent of the seismic activity worldwide takes place in Greece, whereas 50% of the seismic activity in Europe takes place in Greece. It is worth mentioning that the vast majority of the past earthquakes are exhibited in the eastern Mediterranean region. The high seismic activity exhibited in this region is due to the fact that it is located at the boundary of the Africa-Eurasia convergence [58]. Greece often hosts large magnitude earthquakes, whilst a moderate or small magnitude earthquake is felt every two to three days on average. Major shallow earthquakes (M > 8, return period of about 1000 years), which can cause extensive destruction, occur rarely. Although the majority of these earthquakes are shallow, only a few have been recorded as “devastating” for the human environment or for loss of life (e.g., the 1881 Chios, 1953 Cephalonia and 1999 Athens earthquakes). This is due to the fact that the majority of the epicenters of these earthquakes have been located in the sea, and thus, most of the energy released has been effectively dissipated prior to reaching the populated areas.

### 7.5. Intervention Scenarios

In the framework of the principles imposed by past or current regulations and scientific charters (e.g., the Athens Charter 1931 (ICOMOS 1931) and the Venice Charter 1964 (ICOMOS 1964), etc.) [1,2], the key point of all intervention scenarios was the use of compatible and effective, regarding the reduction of the seismic vulnerability, restoration mortars. The restoration mortars were selected through extensive research of available literature [59,60]. The selected mortars were all designed for use in historical buildings through the reverse engineering methodology. The restoration mortars selected exhibit adequate hydraulicity, so as to ensure hardening in high humidity conditions and resilience in the presence of salts. Specifically, two lime-metakaolin and one hydraulic lime mortar and two lime-metakaolin and one hydraulic lime concrete were selected (Table 6). The concretes were designed with the addition of crushed bricks, in order to simulate historical mortars of byzantine monuments that have shown excellent performance in earthquakes [61]. The raw materials used fulfilled all criteria in order to be acceptable for use in historical masonry [62].

**Figure 10:**
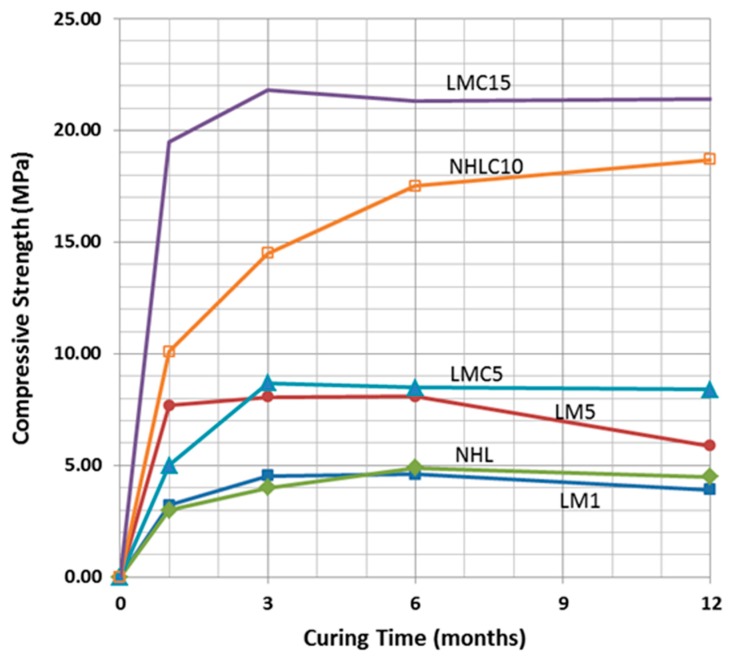
Compressive strength values (MPa) in relation to curing time (months); LM: lime metakaolin mortars, LMC: lime metakaolin concrete, NHL: Natural hydraulic lime mortar, NHLC: Natural hydraulic lime concrete.

The average values of flexural and compressive strength measured for the examined mortars and concretes are listed in Table 7. The flexural strength values result as the average of the value measured for three different prismatic samples for each mortar mix, while the compressive strength values result from the average of the value measured for six different cubic samples for each mortar mix. The selected restoration mortars and concretes achieve a wide range of mechanical strength properties, while exhibiting adequate physicochemical compatibility, especially in the case of the lime-metakaolin mortars. Furthermore, the selected mortars also achieve early compressive strength values (Figure 10).

In the following table (Table 8), the values corresponding to the tangent modulus (static modulus of elasticity, Est) of each mortar/concrete mix examined are presented, as the average estimated for six samples for each mix. In the same table, the values of the dynamic modulus (Ed) for each sample is also presented, as estimated through ultrasonic sound velocity measurements. The tests were conducted on samples that had completed 12 months of curing, so that they were relatively stabilized in terms of the evolution of chemical reactions during hardening.

As expected, the concrete mixes examined presented much higher modulus of elasticity values in comparison to the mortar mixes; this is especially true for the static modulus of elasticity values. Amongst the concretes, the lowest static and dynamic values of elasticity are exhibited by concrete LM_C5_, which also presented the lowest values of flexural and compressive strength; the highest static and dynamic modulus of elasticity values by far are exhibited by the hydraulic lime concrete, although it exhibited lower compressive and flexural strength values than LΜ_C15_ (Table 8). The mortar containing the highest amount of metakaolin per weight. presented the highest compressive strength values and also the highest static modulus of elasticity value. The optimum selection in order to ensure a good behavior under seismic action is the compromise of a high compressive strength and low dynamic elasticity modulus.

### 7.6. Damage Indices

**Figure 11:**
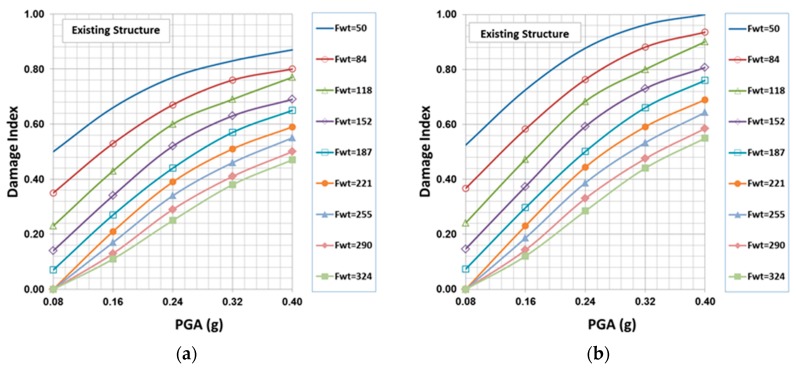

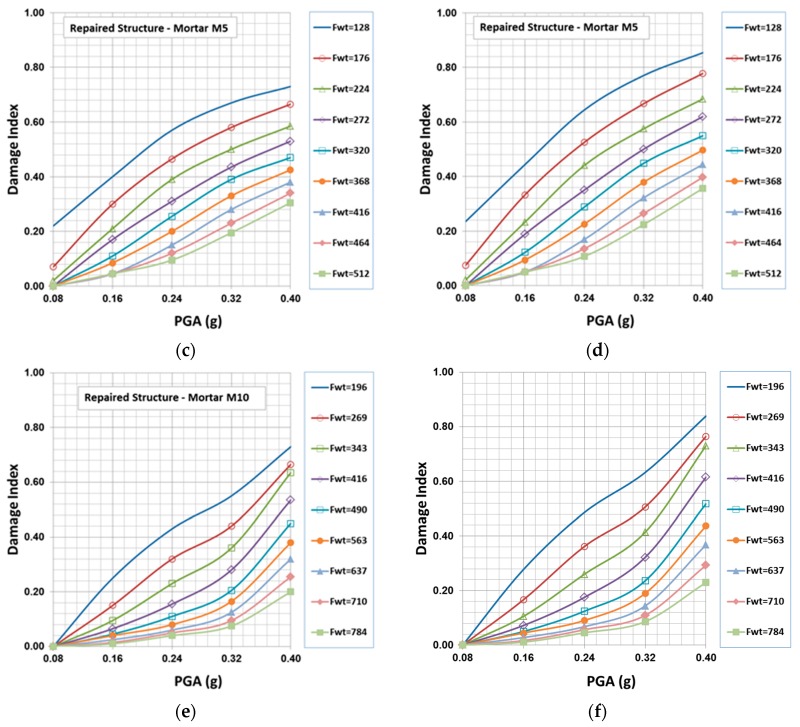
Damage indices for the existing, as well as for the two case of repaired structure with M5 and M10 mortars using the cubic and simplified failure criterion: (**a**) existing structure (cubic); (**b**) existing structure (simplified); (**c**) repaired structure with mortar M5 (cubic); (**d**) repaired structure with mortar M5 (simplified); (**e**) repaired structure with mortar M10 (cubic); (**f**) repaired structure with mortar M10 (simplified).

The failure analysis for the existing structure, as well as for the studied interventions’ scenarios has been based on the failure criteria explained in previous sections. In addition to the main computer program used for the analysis (SAP2000, Computers and Structures, Berkeley, CA), a special computer program, capable of producing a “visual” representation of the failed regions within the structure, has been developed from scratch. The program takes the SAP2000 analysis results as input and gives statistics for the number of failure points for each one of the two proposed failure criteria (Equations (5) and (6)), as well as of the type of failure, providing a general view of the probable damage level and the main type of damages within the structure. Based on these results and using Equation (8), the damage indices (Table 9) have been defined for a range of peak ground accelerations between 0.08 g and 0.40 g and masonry tensile strength ranging from 50 kPa to 324 kPa. Figure 11 shows the damage indices for the existing structure, as well as for the two cases of the repaired structure with M5 and M10 mortars (mortars with compressive strength 5 and 10 MPa respectively).

### 7.7. Fragility Curves

The results concerning the damage indices of the structure were analyzed with probabilistic methods. Especially, the Probability distribution function and the associated probability density function were estimated for each level of peak ground acceleration applied to the structure.

Using these probability distribution functions, the probabilities of structure damage for the three structural performance levels (insignificant, moderate and heavy damage) were determined, and the results, both for existing and repaired structures for the two failure criteria, are presented in Table 10 for the case of the normal distribution.

**Figure 12:**
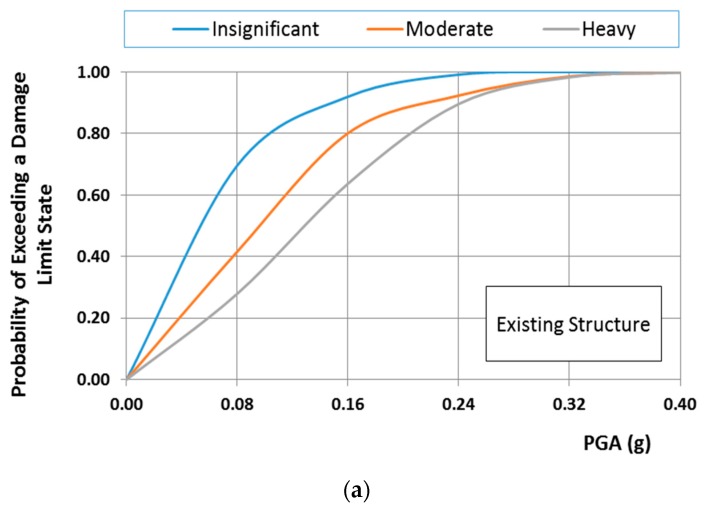

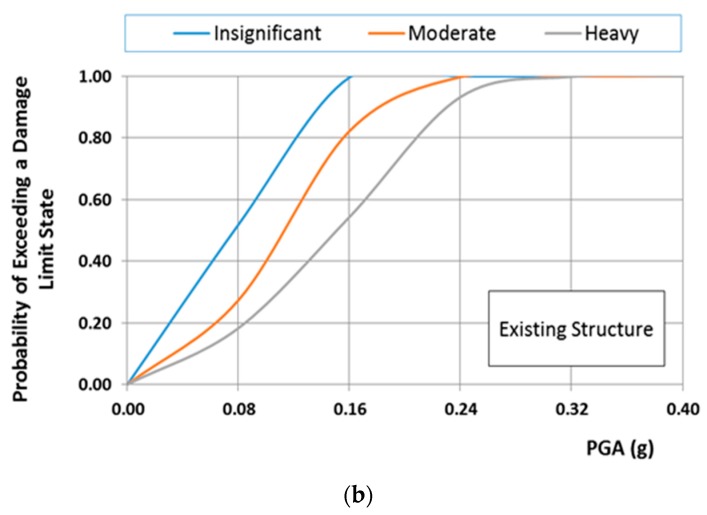
Fragility curves of existing structure normal distribution: (**a**) normal distribution; (**b**) lognormal distribution.

**Figure 13:**
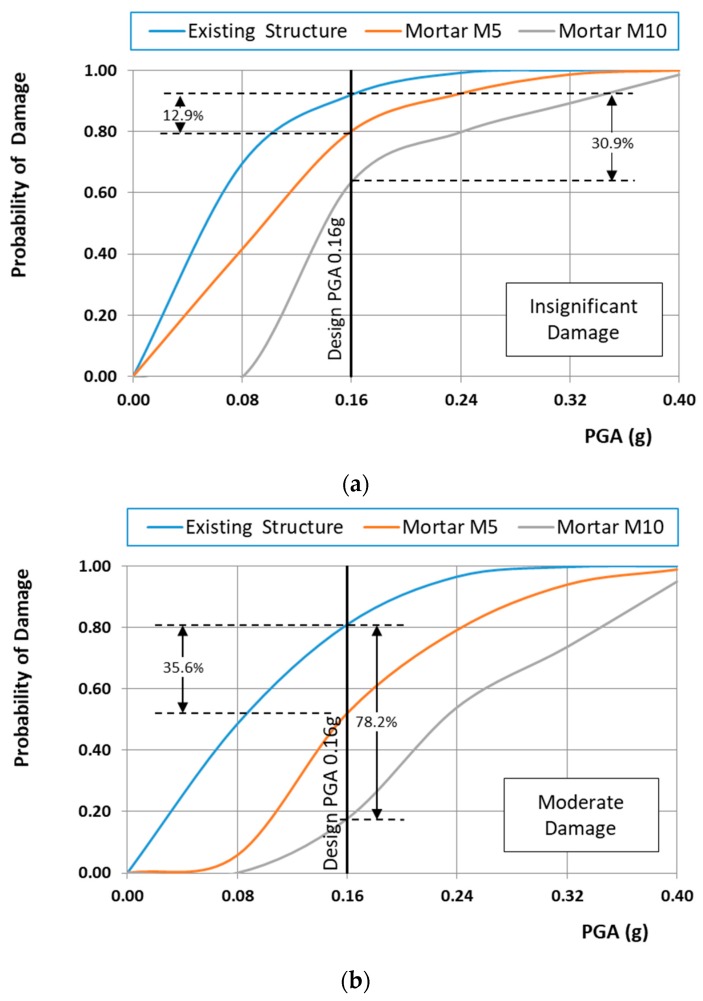

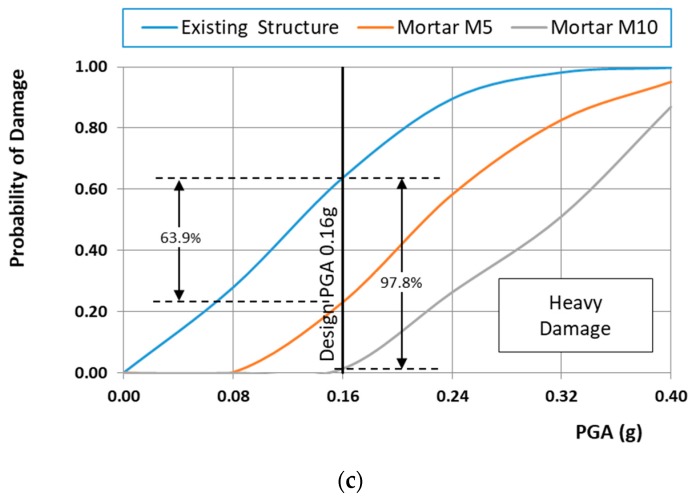
Fragility curves of the structure before and after interventions using the cubic tensor polynomial failure criterion: (**a**) insignificant damage; (**b**) moderate damage; (**c**) heavy damage.

**Figure 14:**
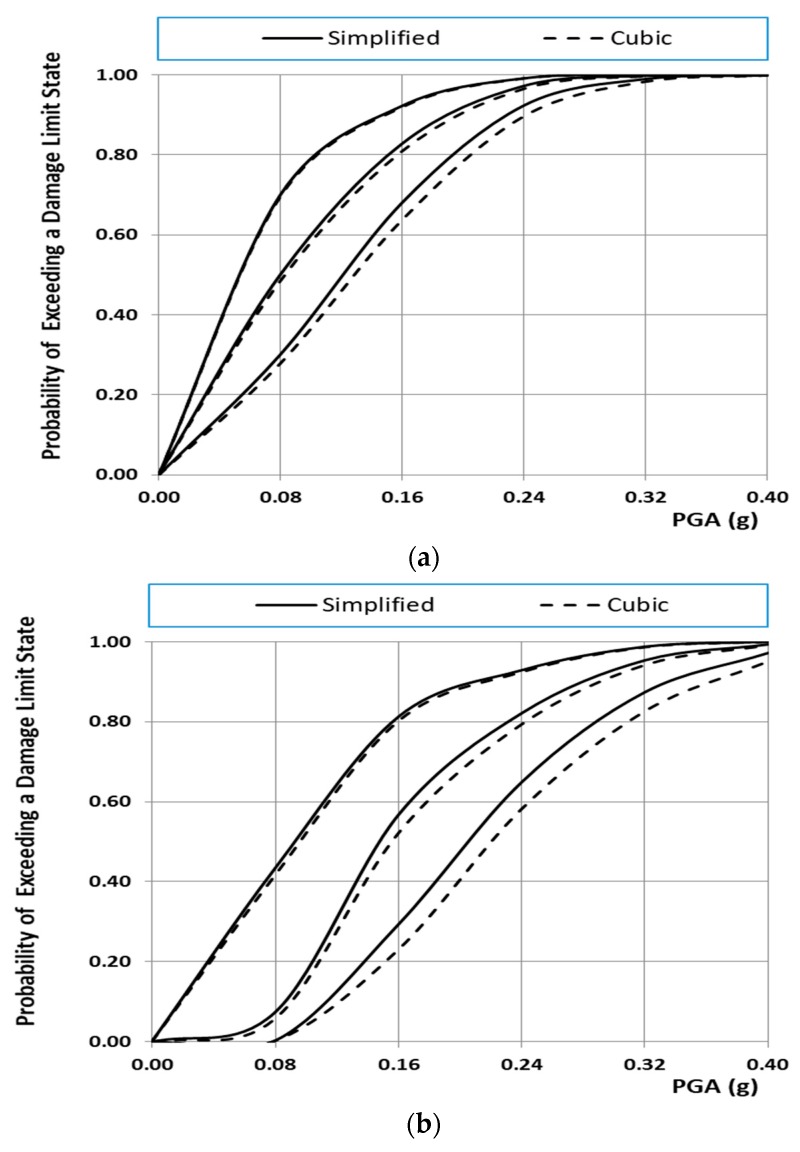

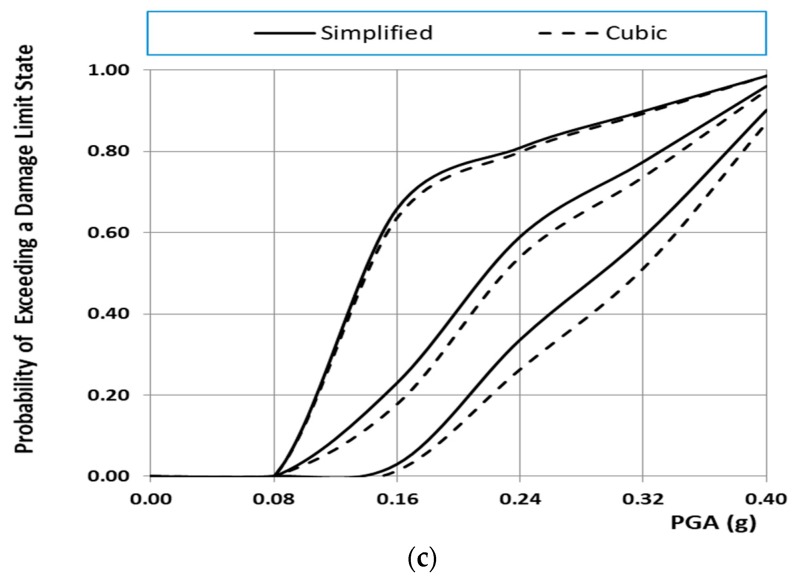
Fragility curves of the existing structure using both failure criteria: (**a**) fragility curves of the existing structure; (**b**) fragility curves of the repaired structure with mortar M5; (**c**) fragility curves of the repaired structure with mortar M10.

Figure 12a,b shows the fragility curves of the existing structure for the normal and lognormal distribution, respectively. From these figures, it is clear that the distribution functions affect crucially the probability of the structure exceeding the damage limit state. Taking into account that the mechanical characteristics of masonry material such as bricks and mortars follow a normal distribution, only fragility curves based on the normal distribution are presented herein. Specifically, Figure 13a–c show the fragility curves of the structure before and after interventions for each on the structural performance level for the case of the normal distribution. These figures show that the fragility curves are important tools in evaluating and ranking the efficiency of the remedial proposals, to address the seismic protection of masonry structural systems. Furthermore, in Figure 14a–c, the seismic vulnerability reduction of the structure for the two rehabilitation scenarios and the two different failure criteria are presented for the case of the use of the normal distribution function.

**Figure 15:**
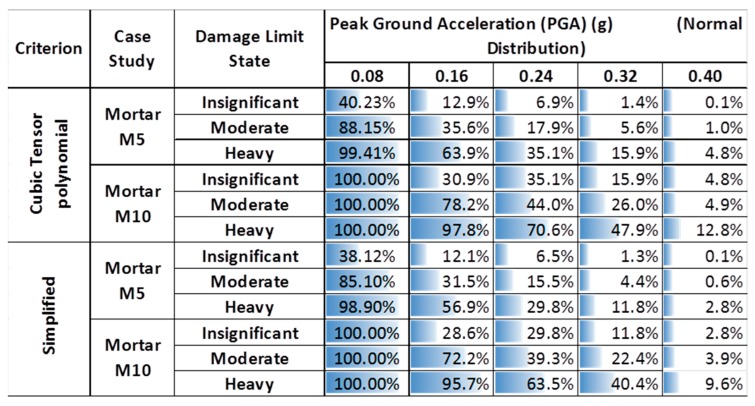
Seismic vulnerability reduction of the structure for the two rehabilitation scenarios (normal distribution).

It should be indicatively mentioned that the probability of heavy damage from a seismic motion with demand represented by PGA = 0.16 g (typical designed value for the monument) is reduced by 63.90% and 97.80% for the repaired structure with restoration mortars M5 and M10, respectively, as can be seen in Figure 13, Figure 14 and Figure 15.

In addition, from Figure 14 and Figure 15, it can be seen that, for the restoration at hand, the effect of the failure criterion is very small despite the fact that the cubic tensor polynomial criterion displays twice the value of the shear strength, as shown in Section 3. This finding should not lead us to the wrong conclusion that the failure criterion does not have an appreciable effect on the outcome. The effect of the failure criterion is very small, as a special case, for the specific restoration under study, which, due to its particular geometrical construction, gave small values for the shear stresses.

The results show that even the use of a restoration mortar exhibiting a compressive strength of 5 MPa can achieve an improvement in terms of damage in the case of an earthquake; the improvement is greater as the damage severity increases. At this point, the restriction deriving from the low compressive strength of the fossiliferous stone must be taken into consideration; NHL_C10_ and LΜ_C15_ are therefore rejected as possible restoration mortars, in order to ensure compatibility with the original structural materials. Thus, restoration mortars LM_5_ and LM_C5_ can be selected to use according to the joint thickness, as these restoration mortars exhibit compatibility with the original structure and serviceability in the environment of the Kaisariani monastery, at the same time contributing to the mechanical performance of the structure under earthquake stresses.

## 8. Conclusions

The vulnerability and assessment, as well as the restoration techniques of historical masonry structures remain considerable challenges from the engineering point view, despite the substantial effort that has taken place in research in the last three decades. In this paper, a new stochastic computational framework for earthquake-resistant design of masonry structural systems has been presented. Namely, fragility analysis has been applied based on the probabilistic behavior of crucial parameters involved in the modelling of the structure, such as the values of materials’ strength and the peak ground acceleration.

According to the analysis of the results for the strengthened structure (which are presented in this paper), it can be concluded that the methodology employed has proven helpful for the modeling and vulnerability assessment of masonry structures, such as historical monuments.

It has been shown that the proposed approach offers a ranking method, which supports civil authorities in optimizing decisions for choosing, among a plethora of structures, which ones present the highest levels of vulnerability and are in need of immediate strengthening. It also helps the practicing engineer to choose the optimal repairing scenario among a number of competing scenarios.

At this point, it should be mentioned as a key point of the conclusions that the final decision for the optimum restoration scenario must be made by a consensual decision-making procedure encompassing the full spectrum of the experts who represent the knowledge disciplines contributing to the solution of the problem at hand. They should take into account the ranking of the effective restoration scenario, based on the proposed methodology, feeling free to investigate a number of ranked scenaria without being obliged to select just the one with the top ranking. The deterministic ranking of the restoration scenario is a useful technique; however, the collective expertise of the experts, which might not be embedded in full in the deterministic scenaria, is considered to be a very significant factor (even more significant than the deterministic rankings) toward a successful proposal for a restoration, even more so if a monument restoration is at hand.

In light of the above, our expressed future goal is the investigation of the effect of the failure criterion used for the estimation of damage indices and their effect on the derived fragility curves. Especially, we will try to study the effect on the results of different failure criteria, both anisotropic and isotropic analytical models, as well as models derived using soft computing techniques, such as artificial neural networks. Furthermore, our salient future research interests include the investigation of using natural fibers as reinforcement in restoration mortars. We will focus on the use of three different natural fibers, such as horsehair, goat hair and straw, as reinforcement in restoration mortars.

## Figures and Tables

**Table 1:** Minima and maxima for both failure criteria/surfaces.

Criteria	Across Axis	Minima			Maxima		
		${\bar{\sigma }}_{x}$	${\bar{\sigma }}_{y}$	$\bar{\tau }$	${\bar{\sigma }}_{x}$	${\bar{\sigma }}_{y}$	$\bar{\tau }$
**Cubic Tensor Failure**	**x**	**−1.39**	−0.72	0.00	**0.48**	−0.44	0.00
	**y**	−0.69	**−1.16**	0.00	−0.25	**0.04**	0.00
	**τ**	−1.17	−1.02	**−0.50**	−1.17	−1.02	**0.50**
**Simplified Failure**	**x**	**−1.41**	−0.74	0.00	**0.45**	−0.42	0.00
	**y**	−0.73	**−1.19**	0.00	−0.25	**0.04**	0.00
	**τ**	−0.50	−0.58	**−0.27**	−0.50	−0.58	**0.27**

**Table 2:** Proposed structural performance levels for un-reinforced masonry. DI, damage index.

Overall Damage	Heavy Damage	Moderate Damage	Insignificant Damage
	Extensive cracking. Face course and veneer may peel off. Noticeable in-plane and out-of-plane offsets.	Extensive cracking. Noticeable in-plane offsets of masonry and minor out-of-plane offsets.	Minor cracking of veneers. Minor spalling in veneers at a few corner openings. No observable out-of-plane offsets.
(DI)	≥25%	15% ≤ ~ < 25%	<15%
	Collapse prevention	Life safety	Immediate occupancy

**Table 3:** Correlation of physicochemical data with tensile strength.

Sample	Inverse Hydraulicity Index (CO_2_/Structurally-Bound Water)	Tensile Strength (MPa)
Sample 1	18.36	0.060
Sample 2	15.48	0.162
Sample 3	9.28	0.381
Sample 4	7.37	0.448

**Table 4:** Rebound hammer test results for the brick elements of the cloisonné.

East Façade Bricks	Average Rebound Number (R)	Standard Deviation	Compressive Strength (MPa)
Brick 1	40.3	2.9	44.14
Brick 2	40.7	2.7	44.82
Brick 3	33.0	1.2	31.09
Brick 4	28.3	1.7	22.64
Brick 5	39.0	1.4	41.76
Brick 6	33.4	1.8	31.80
Brick 7	28.7	1.2	23.38
Brick 8	32.8	1.3	30.64
Brick 9	37.8	3.3	39.54
Brick 10	31.7	0.6	28.72
Brick 11	34.0	1.4	32.87
Brick 12	28.0	0.0	22.19
Brick 13	36.0	1.4	36.43
Brick 14	33.0	0.8	31.09
Brick 15	35.3	1.5	35.09
Brick 16	34.0	0.0	32.87
Brick 17	32.8	1.5	30.64
Brick 18	38.5	1.7	40.88
Brick 19	31.6	1.1	28.60
Brick 20	41.3	1.2	45.92
Brick 21	31.0	1.0	27.53
Brick 22	34.4	1.1	33.58

**Table 5:** Rebound hammer test results for the fossiliferous stone elements of the cloisonné.

East Façade Bricks	Average Rebound Number (R)	Standard Deviation	Compressive Strength (MPa)
Fossiliferous 1	18.8	2.5	8.37
Fossiliferous 2	19.4	1.5	9.14
Fossiliferous 3	20.0	2.0	9.89
Fossiliferous 4	18.2	1.6	7.75
Fossiliferous 5	15.7	1.3	5.24
Fossiliferous 6	18.7	2.6	8.34
Fossiliferous 7	20.0	0.5	9.89
Fossiliferous 8	17.5	0.7	7.00

**Table 6:** Synthesis of selected restoration mortars [59,60].

Sample Code	Lime Powder	Metakaolin	NHL3,5	Silicate Sand (0 to 2 mm)	Silicate Sand (0 to 6 mm)	Crushed Brick (0 to 16 mm)
LΜ_1_	27.5	2.5	-	70.0	-	-
LM_5_	25.0	5.0	-	70.0	-	-
NHL	-	-	25.0	-	75.0	-
LΜ_C5_	27.5	2.5	-	-	35.0	35.0
NHL_C10_	-	-	30.0	-	35.0	35.0
LM_C15_	20.0	10.0	-	-	35.0	35.0

**Table 7:** Flexural and compressive strength values for examined mortar and concrete mixes [59,60].

Sample	Curing Time (Months)	F_f_ (MPa) ^1^	St. Dev. ^3^	F_c_^2^ (MPa)	St. Dev. ^3^
LM_1_	1	1.03	0.29	3.23	0.82
	3	1.20	0.10	4.53	0.18
	6	0.92	0.07	4.60	0.24
	12	1.59	0.34	3.92	1.40
LM_5_	1	2.13	0.21	7.68	0.37
	3	2.13	0.38	8.07	0.27
	6	1.80	0.29	8.09	0.43
	12	1.51	0.46	5.88	0.88
NHL	1	0.70	-	3.00	-
	3	0.70	-	4.00	-
	6	1.00	-	4.90	-
	12	1.00	-	4.50	-
LM_C5_	1	0.90	0.10	5.00	0.15
	3	1.76	0.10	8.70	0.30
	6	1.52	0.41	8.50	0.16
	12	1.50	0.25	8.40	0.36
NHL_C10_	1	0.90	0.47	10.10	0.33
	3	1.82	0.21	14.50	0.84
	6	2.07	0.25	17.50	0.66
	12	2.22	0.27	18.70	0.50
LΜ_C15_	1	2.10	0.29	19.50	0.40
	3	2.36	0.39	21.80	0.63
	6	2.93	0.21	21.30	0.96
	12	2.40	0.35	21.40	1.26

^1^ F_f_: flexural strength (MPa); ^2^ F_c_: compressive strength (MPa); ^3^ St. Dev.: standard deviation.

**Table 8:** Synthesis of selected restoration mortars [59,60].

Sample	Curing Time (Months)	Εst. (MPa)	St. Dev. (MPa)	Εd (MPa)
LΜ_1_	12	300	87	6532
LM_5_	12	336	125	6641
NHL	-	-	-	-
LΜ_C5_	12	824	174	6981
NHL_C10_	12	3574	619	18,547
LM_C15_	12	2419	251	13,334

**Table 9:** Damage Indices for the structure before and after interventions.

Case	Tensile Strength (kPa)	Peak Ground Acceleration (PGA)									
		Cubic Tensor Failure					Simplified Failure				
		0.08	0.16	0.24	0.32	0.4	0.08	0.16	0.24	0.32	0.40
**Existing Structure**	50	0.50	0.66	0.77	0.83	0.87	0.53	0.73	0.88	0.96	1.00
	84	0.35	0.53	0.67	0.76	0.80	0.37	0.58	0.76	0.88	0.94
	118	0.23	0.43	0.60	0.69	0.77	0.24	0.47	0.68	0.80	0.90
	152	0.14	0.34	0.52	0.63	0.69	0.15	0.37	0.59	0.73	0.81
	187	0.07	0.27	0.44	0.57	0.65	0.07	0.30	0.50	0.66	0.76
	221	0.00	0.21	0.39	0.51	0.59	0.00	0.23	0.44	0.59	0.69
	255	0.00	0.17	0.34	0.46	0.55	0.00	0.19	0.39	0.53	0.64
	290	0.00	0.13	0.29	0.41	0.50	0.00	0.14	0.33	0.48	0.59
	324	0.00	0.11	0.25	0.38	0.47	0.00	0.12	0.29	0.44	0.55
**Repaired Structure with Mortar M5**	128	0.22	0.40	0.57	0.67	0.73	0.24	0.44	0.64	0.77	0.85
	176	0.07	0.30	0.47	0.58	0.67	0.07	0.33	0.53	0.67	0.78
	224	0.02	0.21	0.39	0.50	0.59	0.02	0.23	0.44	0.58	0.68
	272	0.00	0.17	0.31	0.44	0.53	0.00	0.19	0.35	0.50	0.62
	320	0.00	0.11	0.26	0.39	0.47	0.00	0.12	0.29	0.45	0.55
	368	0.00	0.09	0.20	0.33	0.43	0.00	0.09	0.23	0.38	0.50
	416	0.00	0.05	0.15	0.28	0.38	0.00	0.05	0.17	0.32	0.44
	464	0.00	0.05	0.12	0.23	0.34	0.00	0.05	0.14	0.26	0.40
	512	0.00	0.05	0.10	0.20	0.31	0.00	0.05	0.11	0.22	0.36
**Repaired Structure with Mortar M10**	196	0.00	0.25	0.43	0.55	0.73	0.00	0.28	0.49	0.63	0.84
	269	0.00	0.15	0.32	0.44	0.67	0.00	0.17	0.36	0.51	0.76
	343	0.00	0.10	0.23	0.36	0.64	0.00	0.11	0.26	0.41	0.73
	416	0.00	0.07	0.16	0.28	0.54	0.00	0.07	0.18	0.32	0.62
	490	0.00	0.05	0.11	0.21	0.45	0.00	0.05	0.12	0.24	0.52
	563	0.00	0.04	0.08	0.17	0.38	0.00	0.04	0.09	0.19	0.44
	637	0.00	0.03	0.06	0.13	0.32	0.00	0.03	0.07	0.14	0.37
	710	0.00	0.02	0.05	0.10	0.26	0.00	0.02	0.06	0.11	0.29
	784	0.00	0.01	0.04	0.08	0.20	0.00	0.01	0.05	0.09	0.23

**Table 10:** Probability of exceeding a damage state for the structure before and after interventions (normal distribution).

Case	Tensile Strength (kPa)	Failure Criterion									
		Cubic Tensor					Simplified				
		Peak Ground Acceleration (PGA)					Peak Ground Acceleration (PGA)				
		0.08	0.16	0.24	0.32	0.40	0.08	0.16	0.24	0.32	0.40
**Existing Structure**	**Insignificant**	0.70	0.92	0.99	1.00	1.00	0.52	1.00	1.00	1.00	1.00
	**Moderate**	0.49	0.81	0.97	1.00	1.00	0.27	0.82	1.00	1.00	1.00
	**Heavy**	0.28	0.64	0.90	0.98	1.00	0.18	0.54	0.93	1.00	1.00
**Repaired Structure with Mortar M5**	**Insignificant**	0.42	0.80	0.92	0.99	1.00	0.19	0.84	0.99	1.00	1.00
	**Moderate**	0.06	0.52	0.79	0.94	0.99	0.03	0.38	0.78	0.99	1.00
	**Heavy**	0.00	0.23	0.58	0.83	0.95	0.01	0.18	0.48	0.83	0.98
**Repaired Structure with Mortar M10**	**Insignificant**	0.07	0.64	0.80	0.89	0.99	0.02	0.49	0.85	0.98	1.00
	**Moderate**	0.00	0.18	0.54	0.74	0.95	0.00	0.14	0.40	0.68	0.99
	**Heavy**	0.00	0.01	0.26	0.51	0.87	0.00	0.06	0.19	0.40	0.88
